# Comparison of Microtensile Bond Strengths of Giomers Using HEMA‐Based and HEMA‐Free Silane Adhesive With Laser‐Cut and Bur‐Cut Dentin

**DOI:** 10.1155/ijod/7335435

**Published:** 2026-09-02

**Authors:** Rania Rashad Omar Omar Taha, Amir Hafez Ibrahim, Shereen Hafez Ibrahim

**Affiliations:** ^1^ Department of Conservative Dentistry, Faculty of Dentistry, Suez University, Suez, Egypt, suezuniv.edu.eg; ^2^ Department of Conservative Dentistry, Faculty of Dentistry, Cairo University, Cairo, Egypt, cu.edu.eg

**Keywords:** acid resistance silane, bioactive, bond strength, bur-cut dentin, ErCr:YSGG laser, HEMA, HEMA-free, laser-cut dentin, universal adhesive

## Abstract

**Objectives:**

Bonding to laser‐cut dentin is challenging to different types of adhesive systems. This study design aimed to compare the microtensile bond strength (µTBS) of surface prereacted glass (S‐PRG)/giomer restoration to both laser‐cut dentin and bur‐cut dentin bonded with either bioactive hydroxyethyle methacrylate (HEMA)‐based adhesive (with S‐PRG filler) or HEMA‐free silane‐based adhesive (without S‐PRG filler) in a laboratory simulation of clinical conditions.

**Materials and Methods:**

Forty molar teeth were divided equally into four groups (*n* = 10 per group): bur‐cut dentin bonded with FL‐Bond II adhesive, laser‐cut dentin bonded with FL‐Bond II, bur‐cut dentin bonded with BeautiBond Xtream universal adhesive, and laser‐cut dentin bonded with BeautiBond Xtream. The dentin was prepared using a bur or laser, depending on the assigned group, after occlusal enamel was removed. After restoration using the respective adhesives, the specimens were thermocycled, their µTBSs were tested using a universal testing machine with a load cell of 100 N and a crosshead speed of 1 mm/min until they failed, and the failure modes were determined using a digital microscope with a 25x magnification. Two‐way ANOVA models were used to statically analyze the collected data. The significance level was set at *p* < 0.05 for all tests. Statistical analysis was performed with R statistical analysis software Version 4.4.2 for Windows.

**Results:**

Two‐way ANOVA showed that the type of bonding agent had a significant effect on bond strength, with the FL‐Bond II samples (16.31 MPa ± 4.10) having significantly higher bond strengths than the BeautiBond Xtream samples (8.58 MPa ± 1.98). The effect of dentin preparation (bur or laser) was not statistically significant (*p* = 0.766). By far the most common failure mode was mixed failure (90%). The bioactive adhesives showed good bond performance regardless of the dentin preparation method; however, the HEMA‐free silane containing universal adhesive was stronger with bur‐cut dentin than laser‐cut dentin, and the HEMA‐based adhesive was stronger than the HEMA‐free adhesive with both types of dentin preparation protocol.

**Conclusions:**

Laser irradiation did not affect the dentin bond strength of resin composites, while the adhesive’s chemical composition played a decisive role in bond performance. The bond performance of FL‐Bond II was superior to BeautiBond Xtream across dentin preparations.

## 1. Introduction

Bonded restoration reclaims cavitated teeth with minimal abrading of the intact tissue. while conserving intact tissue. Bond strength and durability depend on both adhesive‐related factors, such as viscosity, chemical composition, primer chemistry, and pH, and substrate‐related factors, including adhesive viscosity, chemical composition, surface primer chemistry, and pH [1–3]. Dentin can be prepared mechanically with a bur or, increasingly, with erbium lasers; each method produces a distinct smear layer and surface micromorphology that influences bonding [1–3].

Er,Cr:YSGG lasers ablate dentin photothermally with minimal pulpal thermal damage and leave a mica‐like, smear‐layer‐free surface with open dentinal tubules that should, in principle, favor resin infiltration [2, 4–7]. In practice, however, reported microtensile bond strength (µTBS) values for laser‐irradiated dentin relative to bur‐cut dentin range from markedly lower [4, 5], slightly lower [8–12], comparable [8, 9], or even superior [10, 11]—a variability attributed largely to heat‐induced denaturation of the irradiated surface layer, which can impede monomer penetration. Proposed remedies, including removal of the irradiated layer, active adhesive application, chemical surface modification, and adjusted reirradiation parameters [6, 12], have not consistently closed this performance gap [7, 13], marinating on whether the adhesive system itself may be the more decisive variable.

Universal adhesives, the current generation of single‐bottle self‐etch systems, are broadly classed as one‐step (all‐in‐one) or two‐step (separate primer and bonding agent) formulations [9], and their performance is known to vary with viscosity: lower‐viscosity adhesives penetrate more effectively beneath the heat‐affected zone of laser‐cut dentin and form more resin tags within open tubules than higher‐viscosity formulations [14], although manufacturer‐specific differences have made this effect difficult to isolate [15]. Two formulation strategies now aim to improve the durability of this bond specifically at the dentin interface: surface prereacted glass (S‐PRG) fillers, which release fluoride and other ions that promote remineralization and inhibit matrix metalloproteinases [16, 17] and acid‐resistant silane (ARS) technology, recently introduced to reduce the hydrolytic degradation that otherwise compromises silane‐mediated bonding over time [18]. Despite the growing clinical use of Er,Cr:YSGG laser preparation and the introduction of universal adhesives incorporating ARS, no peer‐reviewed data describe how these adhesives perform on laser‐irradiated dentin; existing evidence for one such adhesive, BeautiBond Xtream, is limited to manufacturer‐supplied reports on bur‐cut substrates [19].

This study therefore aimed to evaluate and compare the µTBS of a resin composite to Er,Cr:YSGG laser‐cut and bur‐cut dentin using two adhesives that differ in ARS/S‐PRG content, hydroxyethyle methacrylate (HEMA) content, and step format (one‐step BeautiBond Xtream vs. two‐step FL Bond II). The null hypothesis was that µTBS would not differ significantly between the adhesive systems or between the dentin preparation methods.

## 2. Materials and Methods

In this study, two independent variables were dentin preparation method (bur cutting vs. Er,Cr:YSGG laser irradiation) and adhesive system (FL Bond II vs. BeautiBond Xtream), arranged in a 2 × 2 factorial design across four groups. A light‐cure fluoride‐releasing dental restorative material—Beautifil II LS (Giomer, Shofu Inc., Kyoto, Japan), shade A2created using PRG‐ionomer technology was used throughout. Two bioactive adhesive systems were compared: (a) a two‐step, self‐etch, fluoride‐releasing and recharging, HEMA‐containing bonding system (FL‐Bond II, Shofu Inc.) and (b) a one‐step, HEMA‐free universal adhesive with ARS technology (BeautiBond Xtream, Shofu Inc.). Materials specifications are presented in Table S1.

### 2.1. Study Setting

The study protocol of this experimental laboratory preclinical study was approved by the research ethical committee at the Faculty of Dentistry, October 6 University, Egypt (RECO6U/35‐2022), held on December 12, 2022. The study follows the STROBE guidelines.

### 2.2. The Selection of Teeth and Storage

Following infection control procedures and ethical considerations, this study used 40 noncarious molar teeth that were extracted for periodontal issues from patients aged 40–60 at the oral surgery clinic after obtaining their informed consent. The teeth were then submerged in a 1% chloramine‐T solution for 72 h for disinfection. A × 7 magnification lens was used to inspect the teeth in order to rule out those that had structural flaws or fissures. The selected teeth were meticulously cleaned of tissue accumulation and calculus, polished with pumice, and brushed at a regular speed. The teeth were then kept in an incubator (BTC, model: BT1020, Cairo, Egypt) with distilled water at 37 ± 3°C. Every 5 days, this isolation was changed out. The buccolingual measurements of all the selected teeth at the point of greatest convexity of their crowns were determined to be between 8.4 and 9.4 mm using a digital caliper, and none of them showed cavities or hypoplastic abnormalities.

### 2.3. Sample Size Calculation and Grouping of Teeth

Forty teeth were randomly and equally allocated to four groups of 10 (Group 1: bur‐cut + FL Bond II; Group 2: laser‐cut + FL Bond II; Group 3: bur‐cut + BeautiBond Xtream; and Group 4: laser‐cut + BeautiBond Xtream). Sample size was calculated with 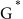
Power (v3.1.9.2) [20] using *α* = 0.05% and 80% power, and an assumed medium effect size (*f* = 0.25), following standard reporting conventions for effect‐size benchmarks.

### 2.4. Teeth Positioning in PMMA Resin‐Blocks and Tooth‐Tissue Preparation

The teeth were embedded in PMMA resin blocks using cylindrical Teflon molds (20 mm internal diameter) and positioned vertically 2.0 mm apically from the cemento‐enamel junction. A dental surveyor ensured proper alignment parallel to each tooth’s long axis, and specimens were stored in distilled water to prevent dehydration. To expose the occlusal flat enamel surface at the level of the main grooves, the occlusal enamel of every tooth was trimmed using a slow‐speed disc (KG Sorensen, Barueri, São Paulo, Brazil) under water cooling. For Groups 1 and 3, residual enamel was then removed with a plain‐cut tungsten carbide fissure bur (KE‐FG057, Kerr, Brea, CA), operated at a rotational speed using a high‐speed handpiece with an air–water spray; the bur was changed every five preparations to avoid dulling and maintain comparable smear‐layer characteristics. The resulting surface roughness was not quantified in this study, which is noted as a limitation. The absence of residual enamel was confirmed by stereomicroscopy (Wild M5A, Heerbrugg, Switzerland). For Groups 2 and 4, dentin was irradiated with a Waterlase iPlus Er,Cr:YSGG laser (Biolase Technology, Irvine, CA) fitted with an 800 µm glass tip, at 8.18 J/cm^2^ energy density, 4.5 W average power, 90 mJ pulse energy, 60 µs pulse duration, 30 Hz repetition rate, and 60% air/80% water spray, in the noncontact mode at a standardized 1.5 mm working distance using a continuous single‐pass mesiodistal scanning technique.

### 2.5. Restoration of Prepared Dentin Surfaces With Resin Composite Materials

Adhesives were applied per manufacturer instructions using a disposable microbrush, actively scrubbed onto dentin with back‐and‐forth strokes for 10 s, air‐thinned for 5 s, and light‐cured for 20 s at zero distance using an LED curing unit (800 mW/cm^2^), and it was actively scrubbed onto the dentin surface using a fully saturated microbrush with back‐and‐forth strokes for 10 s, as recommended by the manufacturer. Thinning with a gentle air stream under mild pressure for 5 s and polymerization for 20 s at zero distance followed. The resin composite was applied in 3–4 increments to a total buildup height of 5–7 mm (Table S1) using a gold‐plated composite applicator (Miltax, Germany), with each increment individually light‐cured.

#### 2.5.1. Thermomechanical Regimen

Specimens underwent 10,000 thermocycles (5–55°C, 25 s dwell/10 s lag per bath, 60 s total cycle) in a thermomechanical chewing simulator (ROBOTA ACH‐09075DCT, Germany) under a 49 N load, approximating 1 year of clinical function and normal chewing force [21].

#### 2.5.2. µTBS Test

Specimens were serially sectioned perpendicular to the bonded surface in both *x* and *y* directions (Isomet 4000, Buehler, Lake Bluff, IL) under water irrigation to produce ~1 mm^2^ cross‐sectional beams. Only central beams were retained (marked prior to sectioning); peripheral beams were excluded due to their susceptibility to stress concentration artifacts and edge defects. A minimum of five beams per tooth were retained for testing, and any beam showing voids, cracks, or an irregular interface on visual inspection was excluded. To select the right bars with the smoothest interfaces for the µTBS test, a visual examination of each beam was then carried out. Each specimen’s cross‐sectional area was measured using a digital caliper (Absolute Digimatic, Mitutoyo, Tokyo, TYO, JPN). A cyanoacrylate glue (Model Repair II Blue, Dentsply‐Sankin, Tokyo, Japan) was used to adhere the samples to a holding device. The beams were then bonded to a jig using a cyanoacrylate adhesive (Model Repair II Blue, Dentsply‐Sankin, Tokyo, Japan) and loaded in tension using a universal testing machine (Instron, MA, USA) with a 100 N load cell at a crosshead speed of 1 mm/min until failure.

#### 2.5.3. Modes of Failure Analysis

Fracture surfaces were examined under a USB digital microscope at ×25 magnification using ViewTi Capture 1.3.0.1 image‐processing software. Failure modes were classified as adhesive (fracture at the resin–dentin interface), cohesive (fracture within the bulk of the dentin or resin composite without exposing the adhesive layer), or mixed (combining both). The proportion of each failure mode was reported as a percentage of the total number of beams per group.

### 2.6. Statistical Analysis

The tooth (*n* = 10/group) was the statistical unit; µTBS values were averaged per tooth before analysis to avoid pseudoreplication from multiple beams per specimen. Normality and variance homogeneity were assessed with the Shapiro–Wilk and Levene tests. A two‐way ANOVA (adhesive system × preparation method as fixed factors) was used, with post hoc simple effects comparisons of estimated marginal means adjusted for multiple comparisons using the false discovery rate method. Effect sizes were calculated per standard conventions. Effect sizes were calculated and interpreted based on standard measures [22, 23]. The significance level was set at *α* = 0.05. All analyses were performed in R v4.4.2 [24].

## 3. Results

The two‐way ANOVA revealed that bonding agent type had a significant and large effect on µTBS (*p* < 0.001, *η* ^2^_*p*_ = 0.61), exceeding the conventional threshold for a large effect (*η* ^2^_*p*_ > 0.14), as shown in Table 1. Dentin preparation method (*p* = 0.766, *η* ^2^_*p*_ < 0.00) and its interaction with bonding agent (*p* = 0.239, *η* ^2^_*p*_ = 0.04, small) were not statistically significant.

**Table 1:** ANOVA test of treatment protocol and the type of bonding agent with sum squares and mean square.

Source	Sum of squares	df	Mean square	*f*‐Value	*p*‐Value	*η* ^2^_*p*_ (95% CI)
Treatment	0.94	1	0.94	0.09	0.766	0.00 (0.00–0.08)
Bonding agent	597.10	1	597.10	56.82	<0.001 ^∗^	0.61 (0.43–0.71)
Treatment × bonding agent	15.07	1	15.07	1.43	0.239	0.04 (0.00–0.18)

*Note: η* ^2^_*p*_, partial eta squared.

Abbreviation: df, degree of freedom.

^∗^Significant (*p* < 0.05).

Regardless of the preparation method, FL Bond II (16.31 ± 4.10 MPa) yielded significantly higher µTBS than BeautiBond Xtream (8.58 ± 1.98 MPa). Simple effects comparisons (Table 2) confirmed this pattern within each preparation subgroup: FL Bond II exceeded BeautiBond Xtream under both laser preparation (16.77 ± 4.68 vs. 7.81 ± 1.92 MPa; *p* < 0.001, *η* ^2^_*p*_ = 0.51, large) and bur preparation (15.84 ± 3.63 vs. 9.35 ± 1.81 MPa; *p* < 0.001, *η* ^2^_*p*_ = 0.36, large). Conversely, the dentin preparation method had no significant effect on µTBS for either BeautiBond Xtream (*p* = 0.297, *η* ^2^_*p*_ = 0.03, small) or FL Bond II (*p* = 0.529, *η* ^2^_*p*_ = 0.01, negligible). The failure mode distributions are summarized in Table 2.

**Table 2:** Simple effects comparisons of estimated marginal means.

Bonding agent	Tensile bond strength (MPa) (mean ± SD)		*f*‐Value	*p*‐Value	*η* ^2^_*p*_ (95% CI)
	Laser	Bur			
BeautiBond Xtream	7.81 ± 1.92	9.35 ± 1.81	1.12	0.297	0.03 (0.00–0.16)
FL Bond	16.77 ± 4.68	15.84 ± 3.63	0.40	0.529	0.01 (0.00–0.12)
*f*‐Value	38.16	20.10	—	—	—
*p*‐Value	<0.001 ^∗^	<0.001 ^∗^	—	—	—
PES (95% CI)	0.51 (0.31–0.64)	0.36 (0.15–0.51)	—	—	—

*Note: η* ^2^_*p*_, partial eta squared.

^∗^Significant (*p* < 0.05).

Mixed failures predominated across all groups: BeautiBond Xtream/laser (mixed 70%, adhesive 22%, and cohesive 8%), BeautiBond Xtream/bur (mixed 72%, adhesive 20%, and cohesive 8%), FL Bond II/laser (mixed 74%, adhesive 10%, and cohesive 16%), and FL Bond II/bur (mixed 76%, adhesive 8%, and cohesive 16%). The trend toward a higher cohesive component in FL Bond II groups is consistent with the greater bulk strength of those bonds, while the higher adhesive component in BeautiBond Xtream groups reflects the comparatively weaker resin–dentin interface produced by that system.

## 4. Discussion

In our study, µTBS remained unaffected by dentin preparation method under 1‐year thermomechanical aging, while adhesive formulation exerted a strong independent effect, with FL Bond II significantly exceeding BeautiBond Xtream irrespective of substrate preparation (*p* < 0.001, *η* ^2^_*p*_ = 0.61). Laser‐ and bur‐prepared dentin exhibited comparable µTBS, corroborating previous reports that Er,Cr:YSGG and related erbium lasers neither improve nor impair bonding relative to conventional bur preparation under comparable conditions [2]. The nonsignificant interaction indicates that the superiority of FL Bond II over BeautiBond Xtream was consistent regardless of dentin preparation method and that neither adhesive was selectively advantaged by laser or bur preparation—the two factors acted independently on µTBS.

Although laser irradiation generates a smear‐free surface with patent dentinal tubules that may facilitate resin infiltration [25], concurrent thermal collagen denaturation can hinder monomer penetration [26, 27], whereas bur preparation produces a smear layer requiring modification by self‐etch adhesives. The absence of significant differences suggests that these opposing substrate effects reached an overall equilibrium, although collagen integrity and mineral alterations were not directly evaluated. In contrast, the adhesive formulation exerted the dominant influence on bond strength. FL Bond II significantly outperformed BeautiBond Xtream [28], likely owing to its two‐step HEMA‐containing chemistry [29], which promotes monomer infiltration and chemical interaction with residual hydroxyapatite [30]. Conversely, the HEMA‐free universal formulation, while designed to reduce hydrolytic degradation and allergenic potential [31–33], has been associated with more permeable hybrid layers [34], reduced polymerization efficiency, and inferior long‐term bond stability [35]. The fluoride‐releasing filler system of FL Bond II may further enhance hybrid‐layer durability through remineralization [36–38], although the individual contributions of HEMA content, filler composition, viscosity, and application protocol cannot be distinguished in the present study. Moreover, all‐in‐one adhesives with greater hydrophilicity could result in weaker, unstable hybrid layers that are more vulnerable to proteolysis and hydrolytic breakdown [39], as was the case with Beautibond Xtreme. Furthermore, it has been noted that all‐in‐one adhesives increased hydrophilicity impairs their capacity to attach, especially to dentin that is naturally hydrophilic, by preventing the acidic monomers in the hybrid layer from fully polymerizing [39].

The large effect size for adhesive type (*η* ^2^_*p*_ = 0.61) indicates that adhesive chemistry was the principal determinant of µTBS, with FL Bond II achieving clinically meaningful improvements that may enhance restoration longevity in stress‐bearing posterior teeth. Conversely, the absence of a preparation method effect supports using Er,Cr:YSGG laser preparation without compromising adhesion while retaining its established benefits of reduced discomfort, greater tissue preservation, and antibacterial potential [12]. Bond strength was assessed using the microtensile test, which minimizes specimen size‐related stress distribution bias and provides greater sensitivity than conventional shear testing [17] because stronger adhesion is expected to better resist the polymerization shrinkage and functional stresses a restoration experiences in service.

## 5. Conclusions

Er,Cr:YSGG laser preparation did not significantly affect µTBS compared to bur cutting, regardless of the adhesive system. FL Bond II demonstrated superior bond performance over BeautiBond Xtream across both preparation methods; however, as the two systems differ across multiple formulation parameters simultaneously, the observed difference should not be attributed to any single factor.

Broader generalizations regarding HEMA‐containing versus HEMA‐free formulations require further comparative studies with controlled single‐variable designs. Despite thermomechanical simulation, the in vitro design cannot fully replicate the harsh oral environment with its biomechanical confounders. Additional limitations include evaluating only a single bur type (tungsten carbide) and a single set of Er,Cr:YSGG irradiation parameters. Further, µTBS was assessed only after thermocycling and was not additionally measured at the conventional 24‐h postbonding timepoint; laser‐induced collagen denaturation was not directly assessed (e.g., by Raman spectroscopy or differential scanning calorimetry); and the disinfection of extracted teeth in chloramine‐T solution and their storage in warm distilled water (37 ± 3°C), while standard laboratory practice, may have altered the collagen structure and hydration state of the dentin and could have influenced the µTBS values obtained.

## Author Contributions

**Rania Rashad Omar Omar Taha and Amir Hafez Ibrahim**: investigation, methodology, writing – original draft. **Shereen Hafez Ibrahim**: conceptualization, methodology, supervision, project administration, writing – review and editing.

## Funding

This study received no institutional funding.

## Disclosure

All authors have read and approved the final and the submitted version of the manuscript. Shereen Hafez Ibrahim had full access to all of the data in this study and takes complete responsibility for the integrity of the data and the accuracy of the data analysis.

## Ethics Statement

The study protocol of this experimental laboratory preclinical study was approved by the research ethical committee at the Faculty of Dentistry, October 6 University, Egypt (RECO6U/35‐2022) at its meeting held on December 12, 2022.

## Consent

Written informed consent with an easy Arabic language as the mother language of participants was signed by the participants to permit the use of their extracted teeth.

## Conflicts of Interest

The authors declare no conflicts of interest.

## Supporting Information

Additional supporting information can be found online in the Supporting Information section.

## Supporting information


**Supporting Information** Table S1: Specifications of the used materials and their biochemical properties.

## Data Availability

The datasets used and analyzed during the current study are available from the corresponding author upon reasonable request.
