# Supplementary material for: Comparison of Microtensile Bond Strengths of Giomers Using HEMA‐Based and HEMA‐Free Silane Adhesive With Laser‐Cut and Bur‐Cut Dentin
Source: Int J Dent. 2026 Sep 2;2026:7335435. doi: 10.1155/ijod/7335435 (PMC13536905; doi:10.1155/ijod/7335435)
Supplement: Supplementary file 1 [file IJOD-2026-7335435-s001.docx]

**Table S1: Materials’ composition, manufacturers and lot number**

| Material | Specification | Composition | Manufacturer |
| --- | --- | --- | --- |
| Beautifil II LS  (shade A2) | A low shrinkage light-cure fluoride releasing dental restorative material (Giomer) created by using pre-reacted glass-ionomer (PRG) technology | Filler:  multifunctional glass filler and S-PRG filler are based on fluoroboroaluminosilicate glass. Filler loading: 81.5wt% (66.3 vol%), particle range: 0.01 to 5μm, mean particle size: 1μm.  Base resin:  Bis-GMA/TEGDMA resin. | Shofu Inc. Kyoto 605-0983, Japan. |
| Beautibond Xtreme | Single bottle self-etch all in one fluoride releasing and recharging light-cure universal adhesive with ARS technology | Adhesive: Bis-GMA,TEGDMA, phosphonic acid monomer, carboxylic acid monomer, acetone, water, acid resistant Silane coupling agent..  pH: 2.3 | Shofu Inc. Kyoto 605-0983, Japan. |
| FL-Bond II | Two bottles, self-etch fluoride releasing and recharging light-cure universal adhesive system | PRIMER A: distilled water, acetone, initiators.  PRIMER B: 2-HEMA, 4-AET, 4-AETA, UDMA, TEGDMA, acetone, initiators, stabilizers, pigment.  BONDING AGENT: distilled water, 2-HEMA, 4-AET, 4-AETA, TEGDMA, UDMA, PRG fillers, DL-camphorquinone, initiators.  pH = 2.4 | Shofu Inc. Kyoto 605-0983, Japan. |
| *4-MET:* 4- Methacryloxy Ethyl Trimellitic anhydride, *UDMA:* Urethane dimethacrylate, *Bis-GMA:* Bisphenol A diglycidylmethacrylate, *Bis-EMA:* Bisphenol A polyethylene glycol diether dimethacrylate, *HEMA:* 2 hydroxyethyl methacrylate, *TEGDMA:* triethyleneglycoldimethacrylate, *4-AET :*4-acrylo ethyl trimillitic acid. | | | |
